# Is repetitive Transcranial Magnetic Stimulation really effective in the treatment of major depression? – Results of a Meta-Analysis

**DOI:** 10.1192/j.eurpsy.2022.767

**Published:** 2022-09-01

**Authors:** S. Kovacs, R. Vida, D. Erdosi, J. Jozwiak-Hagymasy, A. Zemplenyi, T. Tényi, V. Voros, P. Osvath

**Affiliations:** 1 University of Pécs, Faculty Of Pharmacy, Division Of Pharmacoeconomics, Pécs, Hungary; 2 University of Pécs, Faculty Of Pharmacy, Department Of Pharmaceutics, Pécs, Hungary; 3 University of Pécs, Faculty Of Medicine, Department Of Psychiatry And Psychotherapy, Pécs, Hungary

**Keywords:** rTMS, Depression, metaanalysis

## Abstract

**Introduction:**

Clinical studies demonstrated the efficacy of rTMS treatment in major depressive disorder. However, the results of meta-analyses are contradictory due to the heterogeneity of the included studies.

**Objectives:**

The aim was to analyse the effectiveness of rTMS for treatment-resistant major depression.

**Methods:**

A systematic literature review of English-language articles published in the last 10 years was performed on PubMed and Scopus databases according to PRISMA guideline principles. To assess the effects of rTMS on response and remission rates, random-effects model and inverse variance method were used.

**Results:**

23 randomized double-blind sham-controlled studies met the inclusion criteria for quantitative analysis for response (n= 1020 patients) and 12 studies for remission (n= 846 patients). The relative risk for response and remission were 2.19 (95% CI: 1.68-2,86, p=0.000 n=912) and 2.65 (95% CI: 1.32-5,31, p=0.002, n=603), respectively using rTMS as add on treatment (in patients after two antidepressant treatment failures) compared to standard pharmacotherapy. I^2^analysis showed no considerable heterogeneity in the combined effect sizes neither for remission studies (I^2^=23.36%) nor for response studies (I^2^=0.00%).

**Conclusions:**

Transcranial magnetic stimulation became an evidence-based, effective treatment for treatment-resistant major depressive disorder, either as a monotherapy or as an augmentation of pharmacotherapy. However, because of the lack of standardized protocol, a substantial methodological heterogeneity exists. According to our results, rTMS was significantly more effective than sham rTMS in both response and remission outcomes, which is consistent with previous meta-analyses, but the effect size was a bit smaller than what was reported previously.

**Disclosure:**

No significant relationships.

